# Exosomal MicroRNAs as Emerging Liquid Biopsy Biomarkers in Glioma: Diagnostic, Prognostic, and Therapeutic Implications for Neuro-Oncology

**DOI:** 10.7759/cureus.112186

**Published:** 2026-07-06

**Authors:** Waleed Q Bashir, Anam Fatima, Shanza Ali, Shahzeb Ahmad, Fiza Ismail, Muhammad Bilal Yousaf, Ahsan Sarwar, Ahmad Noor, Faiqa I Khan, Haseeb Mehmood Qadri

**Affiliations:** 1 Medicine, Shalamar Medical and Dental College, Lahore, PAK; 2 Neurological Surgery, Punjab Institute of Neurosciences, Lahore, PAK; 3 Gastroenterology and Hepatology, Pakistan Kidney and Liver Institute and Research Center, Lahore, PAK; 4 Medicine, Evercare Hospital, Lahore, PAK; 5 Medicine, Fatima Memorial Hospital College of Medicine and Dentistry, Lahore, PAK; 6 Medicine, Allama Iqbal Medical College, Lahore, PAK; 7 General Surgery, Lahore General Hospital, Lahore, PAK

**Keywords:** biomarkers, brain neoplasms, exosomes, glioblastoma, liquid biopsy, micrornas

## Abstract

Glioblastoma remains the most aggressive malignant brain tumor, with limited survival despite advances in surgery, radiotherapy, and chemotherapy. Current diagnostic modalities are insufficient for early detection and precise monitoring of disease progression, prompting interest in liquid biopsy-based biomarkers. Exosomal microRNAs (miRNAs) have been highlighted as significant biomarkers due to their stability and involvement in tumor progression. This narrative review evaluates current evidence regarding the diagnostic, prognostic, and therapeutic significance of exosomal and extracellular vesicle-associated miRNAs in brain tumors, particularly gliomas. Data were collected from PubMed and Google Scholar, and eight original articles published between 2016 and 2026 were included after careful screening. The findings demonstrated dysregulated expression of exosomal miRNAs, including miR-29b, miR-210, miR-301a, miR-454-3p, and miR-2276-5p, which were significantly associated with tumor grade, recurrence, survival, and treatment response. The majority of studies reported strong diagnostic performance, with receiver operating characteristic/area under the curve values ranging from 0.80 to 0.93, while mechanistic analyses implicated these miRNAs in oncogenic pathways, including PTEN/AKT signaling, hypoxia-mediated progression, autophagy regulation, and angiogenesis. Overall, exosomal miRNAs demonstrate considerable potential as noninvasive biomarkers for prognostication and therapeutic targeting, while their dynamic preoperative alterations further suggest utility in disease monitoring.

## Introduction and background

Gliomas account for more than 70% of primary central nervous system tumors, with glioblastoma (GBM; grade IV glioma) associated with the most aggressive behavior and dismal prognosis [1]. The median survival is reported to be around 15 months, with an annual age-adjusted incidence ranging from 3.19 to 4.17 per 100,000 population [2].

Neuroimaging used for diagnosis includes CT and MRI, followed by histopathological and molecular diagnosis after invasive cranial surgery [3]. However, standard MRI cannot detect lesions smaller than 2-3 mm and cannot be performed continuously; similarly, stereotactic biopsy is invasive and not repeatable [4]. Furthermore, MRI is limited in its ability to differentiate between tumor recurrence and treatment effects [5]. These diagnostic modalities cannot achieve early diagnosis, targeted treatment, and dynamic monitoring [6].

Current treatment strategies include surgical resection, fractionated radiotherapy, and temozolomide (TMZ)-based alkylating chemotherapy [7]. Despite significant advances in these treatment modalities, there is no strong evidence of extended survival among patients [3]. Current strategies also fail to provide insight into molecular evolution in response to therapeutic intervention [8].

These challenges emphasize the importance of a noninvasive modality, such as liquid biopsies, as a diagnostic and monitoring tool for GBM, which involves the identification and quantification of tumor-derived contents [7]. These biopsies are efficient in providing real-time data regarding tumor dynamics, treatment response, and tumor recurrence, overcoming shortcomings of traditional imaging [9]. There are various circulating biomarkers, particularly circulating tumor DNA, circulating microRNA (miRNA), circulating tumor cells, extracellular vesicles (EVs), and circulating nucleosomes [7]. miRNAs are small noncoding RNAs that regulate gene expression at the post-transcriptional level by binding to target mRNA, resulting in translational repression or mRNA degradation. These miRNAs may circulate freely or be encapsulated within exosomes [7].

Exosomes are intraluminal vesicles measuring 50-150 nm in size, containing various biomolecules such as miRNAs, mRNAs, proteins, or lipids, which are protected from enzymatic degradation in the extracellular environment by a surrounding lipid bilayer [7]. Moreover, exosomes can traverse the intact blood-brain barrier, facilitating the release of brain-derived molecular cargo into the peripheral circulation [7]. These have gained significant interest owing to their role in regulating gene expression, cell invasion, cellular proliferation, apoptosis, differentiation, carcinogenesis, and intercellular communication [7,10].

Despite growing research on exosomal RNAs, evidence on exosomal miRNAs in brain tumors remains fragmented due to a lack of consolidated synthesis. Moreover, there is no consensus due to the absence of standardized and validated commercial assays, secondary to variations in sample types, study populations, and study-specific biomarkers. This narrative review summarizes current evidence on exosomal miRNAs as diagnostic, prognostic, and therapeutic biomarkers in brain tumors, particularly gliomas.

## Review

Methodology

This structured narrative review consolidates evidence on the role of exosomal miRNAs as liquid biopsy biomarkers in brain tumors. A comprehensive literature search was conducted by author FI and verified by author HMQ across PubMed and Google Scholar to identify relevant studies published between 2016 and 2026. The search strategy incorporated combinations of the following keywords and Boolean operators: ("microRNA" OR "miRNA" OR "miR") AND ("brain tumor" OR "brain cancer" OR "glioblastoma" OR "glioma") AND ("biomarker" OR "diagnostic" OR "prognostic" OR "therapeutic"). The retrieved articles were manually screened based on predefined inclusion and exclusion criteria.

Eligibility Criteria

Original human studies with histopathologically confirmed brain tumors in which exosomal or EV-associated miRNAs were isolated and analyzed from biological fluids were included. Only full-text articles published in English were included in the analysis. Review articles, letters to editors, animal studies, conference abstracts, and commentaries were excluded.

Data Extraction

A total of 63 articles were identified through PubMed and 88 through Google Scholar. Following the removal of duplicates and screening of titles, abstracts, and full texts according to eligibility criteria, eight studies were included in the final analysis (Table 1). Relevant data were extracted from the included studies, including sample size, tumor type, biofluid source, analyzed miRNA, mechanistic pathway, performance metrics, and diagnostic and prognostic performance. As this was a narrative qualitative review, no formal quantitative analysis was performed. The findings were comparatively analyzed to identify recurring molecular pathways, clinical applications, and emerging trends regarding the diagnostic, prognostic, and therapeutic significance of exosomal miRNAs.

**Table 1:** Summary of primary studies investigating exosomal miRNAs in brain tumors AA: anaplastic astrocytoma; AUC: area under the curve; DFS: disease-free survival; EV: extracellular vesicle; GBM: glioblastoma; KPS: Karnofsky Performance Status; miRNA: microRNA; NTA: nanoparticle tracking analysis; OS: overall survival; ROC: receiver operating characteristic; TEM: transmission electron microscopy

Author	Sample size	Biofluid/EV source	Tumor type/grade	Exosome/miRNA	Detection method	Biomarker performance metrics	Mechanistic/functional findings	Expression pattern	Clinical implications
Lan et al. [1]	Glioma (60), Control (43)	Serum EVs	Glioma (GBM Grade IV, pilocytic astrocytoma, diffuse astrocytoma, anaplastic astrocytoma)	miR-301a	qRT-PCR	AUC = 0.937	Activated AKT/FAK via PTEN suppression; cell proliferation and invasion assays	Upregulated in gliomas; associated with lower KPS score and overall survival	Diagnostic and prognostic biomarker for high-grade GBM
Geng et al. [8]	-	CSF EVs; tumor tissue	GBM	miR-9	qRT-PCR; TEM + NTA analysis	AUC = 0.800	miR-9/DACT3 axis promotes migration and growth in malignant phenotype; cell viability, migration, and wound-healing assays	Upregulated in glioma patients	Diagnostic biomarker
Shao et al. [11]	GBM (24), Control (24)	Serum exosomes and tumor tissue	Glioma (Grade II, III, IV; GBM)	miR-454-3p	qRT-PCR	AUC = 0.8663	Suppresses autophagy by targeting ATG12; inhibits cell proliferation, migration, and invasion	Downregulated in tumor tissue, upregulated in exosomes; associated with poor prognosis	Diagnostic and prognostic biomarker
Zhong et al. [12]	GBM (107), AA (40), Control (80)	Serum exosomes	GBM (Grade IV), anaplastic astrocytoma (Grade III)	miR-29b	qRT-PCR	AUC = 0.866	-	Downregulated in GBM vs AA and controls; low levels associated with shorter OS and DFS; increased post-treatment	Diagnostic, prognostic, and treatment-monitoring biomarker
Lan et al. [13]	Glioma (91), Control (50)	Serum exosomes	Glioma	miR-210	qRT-PCR	AUC = 0.856	Linked with HIF-1α hypoxia signaling	Upregulated; associated with tumor grade; downregulated after surgery; reflects hypoxic tumor microenvironment	Diagnostic and prognostic biomarker; hypoxia indicator
Tzaridis et al. [14]	GBM (55), Control (10)	Serum EVs	GBM	miR-15b-3p, miR-21-3p, miR-106-5p, miR-328-3p	qRT-PCR; CD44 immunoprecipitation	-	CD44 enrichment enhances tumor specificity	miR-21-3p and miR-106a-5p upregulated in GBM; miR-15b-3p associated with overall survival	Prognostic biomarker
Sun et al. [15]	Glioma (124), Control (36)	Plasma exosomes	Glioma	miR-2276-5p	qRT-PCR	AUC = 0.8107	Potentially targets the RAB13 gene	Downregulated in glioma; associated with poor survival	Diagnostic and prognostic biomarker
Simionescu et al. [16]	Pre/post-op GBM patients	Serum EVs	GBM	miR-106-5p, miR-766-3p, miR-486-3p, miR-30d-5p, miR-625-5p	qRT-PCR	-	-	miR-625-5p detected only preoperatively; miR-30d-5p downregulated after surgery; others remained elevated	Biomarker for disease regression and recurrence monitoring

Overview of key findings

Exosomal and EV miRNAs were isolated from serum, tumor tissue, and CSF of mainly glioma and GBM patients. qRT-PCR was uniformly used for miRNA detection across all studies. A distinct pattern of dysregulation was observed, with several miRNAs upregulated, including miR-301a, miR-454-3p, miR-210, miR-9, miR-21-3p, and miR-106a-5p. Other miRNAs were downregulated, including miR-29b, miR-625-5p, and miR-2276-5p. The expression changes across studies were associated with variables including tumor grade, overall survival, diagnostic utility, disease progression, postoperative reduction, and recurrence assessment.

The majority of miRNAs demonstrated good diagnostic accuracy, with receiver operating characteristic/area under the curve values ranging from 0.80 to 0.93, highlighting the diagnostic utility of exosomal miRNAs in distinguishing glioma patients from healthy individuals or patients with low-grade gliomas.

Functional and mechanistic analyses demonstrated that exosomal miRNAs contribute to glioma progression through the regulation of major tumor-associated pathways, including PTEN/AKT/FAK signaling, hypoxia-driven HIF-1α activation, and ATG12-mediated autophagy. The RAB13-associated tumor aggressiveness and the miR-9/DACT3 pathway are involved in cellular proliferation, migration, and invasion.

Discussion

The present review demonstrates accumulating evidence supporting exosomal and EV miRNAs as minimally invasive biomarkers in glioma patients. A wide range of circulating biomarkers, including tumor-derived cells, nucleic acids, and proteins, have been reported in the blood of glioma patients. However, exosomal miRNAs have emerged as promising biomarkers due to their clear differential expression profiles between glioma patients and healthy controls [17].

Diagnostic Value

Across the included studies, several exosomal miRNAs demonstrate consistent dysregulation in tumor patients compared with healthy controls, which is consistent with current literature highlighting their role in disease progression, immune modulation, angiogenesis, and immune suppression within the glioma microenvironment [18]. In our study, the glioma cohort reported diagnostic AUCs for exosomal miRNAs in the range of 0.80-0.93, indicating high diagnostic accuracy and supporting the growing evidence of exosomal miRNAs as noninvasive diagnostic biomarkers in brain tumors.

Exosomal miRNAs are small and easily amplified via PCR, making them more suitable than exosomal proteins, which are not only less abundant but also more difficult to quantify. In our study, the majority of exosomes were obtained from serum samples; however, the literature suggests that CSF-derived exosomes carry more tumor-specific miRNAs, as serum miRNA levels can be diluted by non-tumor sources [4].

**Figure 1:**
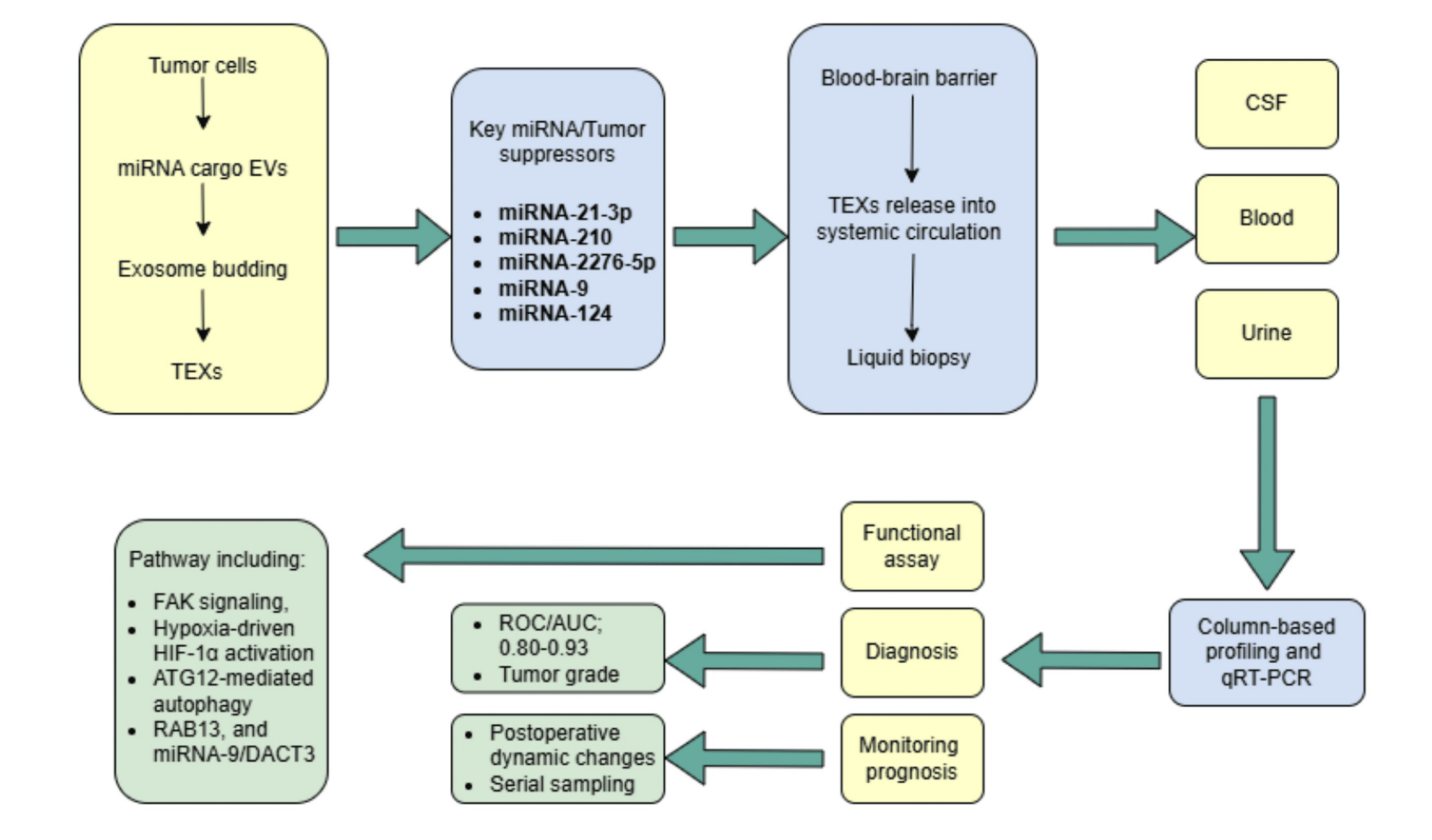
Author-proposed illustration of tumor-derived exosomes carrying key miRNAs in glioma patients and their applications in diagnosis, prognosis, and therapeutic monitoring through liquid biopsy AUC: area under the curve; EV: extracellular vesicle; HIF-1α: hypoxia-inducible factor 1 alpha; miRNA: microRNA; ROC: receiver operating characteristic; TEX: tumor-derived exosome This figure was created using Microsoft PowerPoint 365 (Microsoft Corporation, Redmond, WA, USA).

In addition, the literature highlights upregulation of miRNAs, including miR-222, miR-9, and miR-26a, in signaling pathways that promote tumor growth [19]. Common diagnostic markers include miR-21-3p and miR-106a-5p, which are upregulated in GBM patients [1]. Furthermore, miRNAs provide longitudinal utility for tracking tumor burden over time, as highlighted by a postoperative drop in levels of exosomal miR-301a and miR-210, which then rose with relapse in a study by Lan et al. (Figure 1) [1,3].

Prognostic Significance

In this review, different studies highlighted specific miRNAs. High levels of exosomal miR-454-3p have been associated with poor prognosis, along with exosomal miR-301a, which shows a negative correlation with patients’ KPS scores [1]. Additionally, miRNAs have the potential to reflect tumor grade, with higher levels of exosomal miR-210 being associated with higher tumor grade [13]. Low expression of miR-2276-5p in glioma patients is associated with poor patient survival [15]. Similarly, Tzaridis et al. highlighted a prognostic miRNA panel comprising miR-15b-3p, miR-21-3p, miR-106a-5p, and miR-328-3p, which demonstrated potential in predicting clinical outcomes in patients with glioma [14].

Therapeutic Implications

Exosomes function as critical mediators of intercellular communication within the tumor microenvironment by transferring miRNAs, proteins, and other bioactive molecules between tumor cells and surrounding stromal, immune, and endothelial cells [18]. Hannafon and Ding highlight that exosomal miRNAs can induce permanent changes in recipient cells by mediating gene expression and secreting matrix metalloproteinases, which facilitate tumor cell migration and transport oncogenic and proangiogenic factors, promoting premetastatic niche formation [20].

As highlighted in this review, functional and mechanistic analyses across the included studies demonstrate that exosomal miRNAs are not merely passive biomarkers but active regulators of tumor progression. The identified miRNAs are involved in key oncogenic pathways associated with proliferation, invasion, migration, autophagy, and therapeutic resistance. miR-301a findings are consistent with existing literature, which highlights its secretion by hypoxic GBM cells (located in the tumor core) and its downregulation of TCEAL7 upon internalization, ultimately contributing to increased resistance to radiation-induced DNA damage [19]. Regarding chemotherapy, exosomal miRNAs can contribute to resistance to TMZ (a standard chemotherapeutic agent for GBM). MGMT-mediated DNA repair can further reduce chemotherapy effectiveness [21]. Similarly, in this study, upregulation of miR-210 was shown to contribute to GBM aggressiveness and treatment resistance [13]. miR-21 was also associated with the VEGF signaling pathway, promoting tumor angiogenesis and supporting tumor proliferation despite stress induced by radiotherapy or chemotherapy [19]. Similarly, by targeting the GSK3β pathway, miR-26a has been shown to enhance cellular capacity for DNA strand-break repair, further highlighting the role of miRNAs in both protecting and supporting tumor cell survival [22]. Conversely, miR-135b has been shown to suppress tumorigenesis in GBM stem-like cells, leading to reduced proliferation and migration [23]. Collectively, these findings suggest that exosomal miRNAs may contribute to GBM treatment failure by enhancing tumor survival during chemotherapy and radiotherapy.

Thus, miRNAs may enable earlier detection than imaging, allow repeat sampling, and reflect functional tumor activity rather than only genetic mutations, with levels shifting rapidly in response to environmental pressures such as hypoxia or therapeutic stress [20].

Clinical Translational Challenges

A major factor reducing treatment efficacy is the heterogeneous permeability of the BBB, blood-tumor barrier, and tumor microenvironment, which complicates drug delivery to the tumor [24]. Additionally, current evidence is predominantly based on preclinical and small-scale studies; therefore, the field lacks clinical translation. These clinical translational challenges may hinder further reliable research into the use of miRNAs in this context.

Limitations

Considerable heterogeneity existed among the included studies in terms of tumor types, glioma grades, and sampling fluids. Each study investigated a different exosomal miRNA. The association of each exosomal miRNA with malignancies from other organ systems further limited its specificity and intended clinical role as an early screening biomarker for glioma, resulting in a lack of standardization in biomarker classification. This limits the establishment of a clinically applicable conclusion.

Recommendations

Future research should prioritize glioma-specific miRNAs with diagnostic accuracy and reproducibility across different glioma grades and body fluids. Further investigation of individuals with familial patterns of glioma, focusing on genetic, epigenetic, transcriptomic, and immune mechanisms involved in tumor progression, may help establish reliable biomarkers for early detection and screening of glioma.

## Conclusions

The strong ROC/AUC performance of exosomal miRNAs across analyzed studies as liquid biopsy biomarkers in GBM demonstrates high diagnostic accuracy, prognostic value, utility in recurrence surveillance, and longitudinal disease monitoring, along with associations with tumor grade, survival outcomes, and postoperative tumor dynamics. Furthermore, mechanistic analyses highlight the involvement of miRNAs in key oncogenic pathways related to hypoxia, invasion, proliferation, autophagy, and therapeutic resistance, reinforcing their central role in glioma biology.
